# LRP5 and LRP6 Are Not Required for Protective Antigen–Mediated Internalization or Lethality of Anthrax Lethal Toxin

**DOI:** 10.1371/journal.ppat.0030027

**Published:** 2007-03-02

**Authors:** John J Young, Jennifer L Bromberg-White, Cassandra Zylstra, Joseph T Church, Elissa Boguslawski, James H Resau, Bart O Williams, Nicholas S Duesbery

**Affiliations:** 1 Laboratory of Cancer and Developmental Cell Biology, Van Andel Research Institute, Grand Rapids, Michigan, United States of America; 2 Laboratory of Cell Signaling and Carcinogenesis, Van Andel Research Institute, Grand Rapids, Michigan, United States of America; 3 Laboratory of Analytical, Cellular, and Molecular Microscopy, Van Andel Research Institute, Grand Rapids, Michigan, United States of America; The Salk Institute for Biological Studies, United States of America

## Abstract

Anthrax toxin (AnTx) plays a key role in the pathogenesis of anthrax. AnTx is composed of three proteins: protective antigen (PA), edema factor, and lethal factor (LF). PA is not toxic but serves to bind cells and translocate the toxic edema factor or LF moieties to the cytosol. Recently, the low-density lipoprotein receptor–related protein LRP6 has been reported to mediate internalization and lethality of AnTx. Based on its similarity to LRP6, we hypothesized that LRP5 may also play a role in cellular uptake of AnTx. We assayed PA-dependent uptake of anthrax LF or a cytotoxic LF fusion protein (FP59) in cells and mice harboring targeted deletions of *Lrp5* or *Lrp6*. Unexpectedly, we observed that uptake was unaltered in the presence or absence of either Lrp5 or Lrp6 expression. Moreover, we observed efficient PA-mediated uptake into anthrax toxin receptor (ANTXR)–deficient Chinese hamster ovary cells (PR230) that had been stably engineered to express either human *ANTXR1* or human *ANTXR2* in the presence or absence of siRNA specific for LRP5 or LRP6. Our results demonstrate that neither LRP5 nor LRP6 is necessary for PA-mediated internalization or lethality of anthrax lethal toxin.

## Author Summary

The effects of many pathogenic bacteria are caused by the toxins they release. The toxin released by bacteria that cause anthrax is particularly fascinating since it is made of three different proteins: edema factor, lethal factor, and protective antigen (PA). On their own, each of these proteins is harmless, but when combined, they are deadly. This is because edema factor and lethal factor can exert their poisonous effects only after they have been moved into cells by PA. Determining exactly how PA does this is seen as a critical step in developing medicines that will fight anthrax. That is why a recent report suggesting that LRP6, an outer cell protein, was needed for PA to move the other toxin proteins into cells, was greeted with such interest. However, we now show that mice or cells lacking LRP6, or a related protein called LRP5, are still susceptible to anthrax toxin. The discovery that PA can move lethal factor and edema factor into cells without the help of LRP6 presents a significant challenge to the previously published model. These findings will help focus the efforts of scientists working on new ways to treat anthrax.

## Introduction

Anthrax is caused by a large, Gram-positive bacteria called Bacillus anthracis. Early work with animal models for anthrax established a central role for an exotoxin (anthrax toxin [AnTx]) in its pathogenesis [1,2]. AnTx is comprised of three proteins: protective antigen (PA), edema factor (EF), and lethal factor (LF). PA, by itself, is not toxic. Instead, it serves to translocate EF or LF to the cytosol [3–6]. LF is a Zn^2+^-metalloprotease which specifically cleaves the NH_2_-termini of mitogen-activated protein kinase kinases (MKKs) 1–4, 6, and 7, but not MKK 5 [7–10], resulting in their inactivation [7,11,12]. Combinations of PA plus EF or LF are respectively referred to as edema toxin or lethal toxin (LeTx) [13]. In vitro, treatment of some murine macrophage–derived cell lines with LeTx causes abrupt cell death by lysis [14] (see Video S1).

Two cell surface receptors for PA (anthrax toxin receptor [ANTXR] 1 and 2) have been identified [15,16]. Following binding to ANTXR, PA is cleaved by cell surface–associated furin, removing a 20-kDa fragment and leaving a 63-kDa fragment attached to the receptor. This step is necessary to reveal a binding site for EF or LF [17], as well as to remove steric hindrances to its subsequent oligomerization into a heptamer [6,18,19]. Following this, the toxin complex internalizes via the endosomal pathway [14,20,21].

Using an expressed sequence tag (EST) screen to inactivate chromosomal genes, Wei et al. [22] recently reported that LRP6 plays an essential role in PA-dependent internalization of anthrax LF. LRP6 and the closely related LRP5 are grouped together as a distinct subfamily of low-density lipoprotein (LDL) receptor–like proteins, based upon their common distinctive extracellular structure consisting of four repeats of a prototypic YWTD β-propeller-EGF-like domain [23–25]. They are co-receptors for Wnt ligands, which is a large family of secreted glycoproteins that initiate signaling by binding to members of the Frizzled family of seven transmembrane receptors [26]. Loss-of-function mutations in mice have shown the importance of Wnt molecules in the development of numerous tissues and organs [27]. *Lrp6*-deficient mice display phenotypes similar to, but not as severe as, those seen in several Wnt gene knockouts, and die between embryonic day 14.5 and birth [28]. The *Drosophila* homolog of *LRP5* and *LRP6, arrow,* is required for Wnt signaling in the fly, and loss of *arrow* phenocopies loss of *wingless (wg)* [29]. Mice homozygous for an allele of *Lrp5* encoding a truncated version of the protein recapitulate features of the autosomal recessive human disorder osteoporosis pseudoglioma syndrome (OPPG) [30,31]. Patients with OPPG have both a markedly decreased bone mineral density and persistence of the embryonic hyaloid vascular system [32–35]. Mutations that inactivate the *LRP5* gene in humans cause OPPG [36]. Further confirming the importance of LRP5 in accruing normal bone mass, families with an autosomal dominant syndrome characterized by extremely high bone mineral density have gain-of-function point mutations in *LRP5* [37–40]. In addition, mice engineered to express a point mutant of *Lrp5* associated with high bone mass in humans also develop high bone mass [41].

Based on the related structure and function of LRP6 and LRP5 in Wnt signaling, we reasoned that LRP5 might also play a role in PA-mediated toxicity. To test this, we assayed PA-mediated uptake of LF or FP59, a chimeric toxin consisting of the amino-terminus of LF fused with *Pseudomonas* exotoxin A, in vitro and in vivo using cells and mice harboring targeted deletions of *Lrp5*. In both cases, PA mediated efficient delivery of toxin. Unexpectedly, similar results were obtained for mice harboring targeted deletions of *Lrp6*. Contrary to a previous report, our results demonstrate that neither LRP5 nor LRP6 is necessary for PA-mediated internalization or lethality of anthrax LeTx.

## Results

### Neither Loss of *Lrp5* Expression nor Heterozygous Expression of *Lrp6* Impairs LeTx Lethality In Vivo

**Figure 1:**
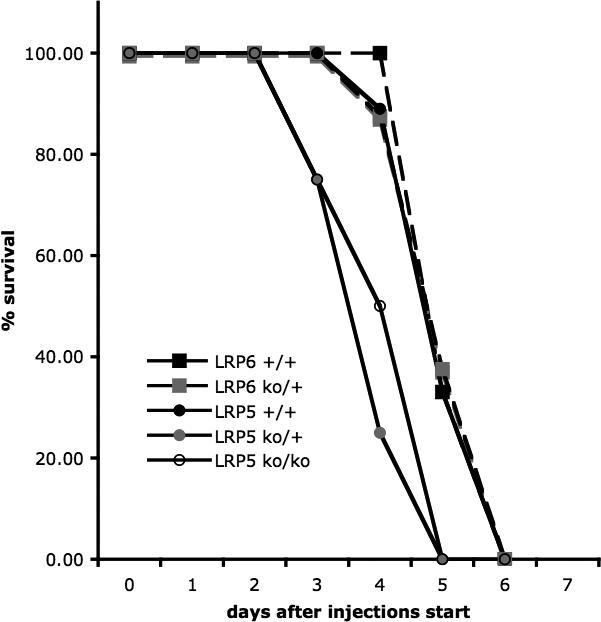
LeTx Challenge of LRP5- and LRP6-Deficient Mice To assess the effects of LRP5 or LRP6 deficiency upon LeTx sensitivity in vivo, LRP5^+/+^ parental (*n* = 9), LRP5^+/−^ heterozygous (*n* = 4), and LRP5^−/−^ nullizygous (*n* = 4) mice, as well as LRP6^+/+^ parental (*n* = 6) and LRP6^+/−^ heterozygous (*n* = 8) mice, were injected daily via the tail vein with 50 μl of neutral buffered saline containing PA (50 ug) and lethal factor (10 ug). The percentage of mice surviving treatment is plotted versus the numbers of days after the mice first received an injection of LeTx. ko/ko, homozygous for knockout allele; ko/+, heterozygous for the knockout allele and the wild-type allele; +/+, homozygous for the wild-type allele.

Wei et al. [22] reported that a polyclonal antibody raised against LRP6 could protect cells from killing by LeTx. Based on this result, the authors suggested that the immunological targeting of LRP6 may prove useful in protecting against the effects of accumulated toxin during the late stages of anthrax disease when antibacterial methods normally are no longer of therapeutic value. To test this hypothesis, we challenged mice having targeted deletions of *Lrp5* [42] or *Lrp6* [28] with daily intravenous injections of anthrax LeTx (50 ug of PA and 10 ug of LF). Previous work in our lab with athymic nude mice on a BALB/cJ background has shown that this dose of LeTx is sufficient to cause hypotensive shock leading to death within 6 d (unpublished data). *Lrp6* ^−/−^ mice were not used in this experiment since they do not survive to birth [28]. Regardless of the status of *Lrp5* or *Lrp6* expression, mice injected with LeTx died within 6 d of the start of treatment (Figure 1). These results indicate that neither loss of *Lrp5* expression nor heterozygous expression of *Lrp6* impairs LeTx lethality.

### Neither *Lrp5* nor *Lrp6* Is Essential for PA-Mediated Uptake of FP59 or LF In Vitro

**Figure 2:**
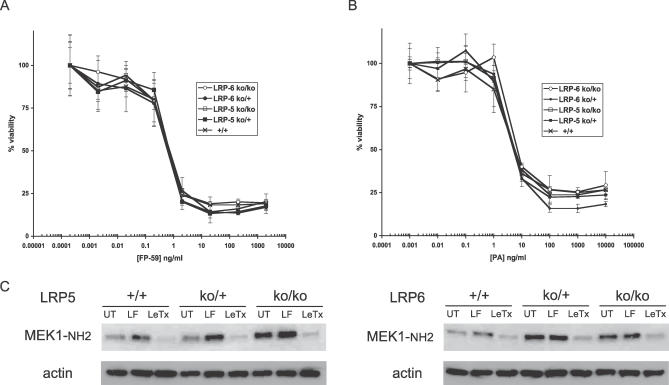
In Vitro Treatment of Embryonic Fibroblasts from LRP5- and LRP6-Deficient Mice with PA Plus FP59 or LF (A) To assess the effects of LRP5 or LRP6 deficiency upon LeTx-sensitivity in vitro, embryonic fibroblasts from LRP5 and LRP6 parental (+/+), heterozygous (ko/+), and nullizygous (ko/ko) mice were treated with PA (1 ug/ml) and FP59 (20 pg/ml–2 ug/ml). Cell viability (oordinate) at the end of 24 h of incubation is plotted versus the concentration of FP59 (abcissa). Error bars indicate standard deviation between three replicates in a single experiment. (B) Alternatively, embryonic fibroblasts from the same mice were treated with FP59 (1 ng/ml) and PA (1 pg/ml–10 ug/ml). Cell viability (oordinate) at the end of 24 h of incubation is plotted versus the concentration of PA (abcissa). Error bars indicate standard deviation between three replicates in a single experiment. This plot is representative of three independent experiments. (C) As an independent indicator of PA entry into embryonic fibroblasts derived from mice with targeted deletions of LRP5 (upper panel) and LRP6 (lower panel), cleavage of MEK1 was assessed by immunoblotting with antibodies that are specific for the NH_2_-terminus of MEK1. UT, untreated.

Wei et al. [22] observed that antisense expression of an EST corresponding to an intronic sequence between exons 21 and 22 of the *LRP6* gene could 1) reduce expression of *LRP6,* and 2) protect M2182 prostate carcinoma cells from PA-mediated uptake of FP59, a cytotoxic fusion protein consisting of the N-terminus of LF genetically fused with the ADP-ribosylating domain of *Pseudomonas* exotoxin A [43]. These observations formed the basis for their conclusion that LRP6 was essential for PA-dependent uptake into cells. To test whether Lrp5 was essential for PA-mediated internalization, we isolated murine embryonic fibroblasts (MEFs) from *Lrp5* ^+/+^ parental, *Lrp5* ^+/−^ heterozygous, and *Lrp5* ^−/−^ nullizygous mice, and treated them with PA plus FP59. *Lrp5* ^+/+^ parental, *Lrp5* ^+/−^ heterozygous, and *Lrp5^−/−^* nullizygous MEFs demonstrated equal sensitivity to treatment with a constant amount of PA plus varying concentrations of FP59 (Figure 2A), or with a varying amount of PA plus constant concentrations of FP59 (Figure 2B). These results indicate that loss of *Lrp5* expression alters neither MEF sensitivity to FP59 nor the ability of PA to translocate FP59 into cells. As an independent measure of PA-mediated entry, MEFs were treated with PA plus LF, and lysates were immunoblotted for N-terminal proteolysis of mitogen activated protein kinase/extracellular signal-regulated kinase kinase (MEK) 1. *Lrp5* ^+/+^ parental, *Lrp5* ^+/−^ heterozygous, and *Lrp5* ^−/−^ nullizygous MEFs demonstrated equal cleavage of MEK1 following treatment with PA plus LF (Figure 2C). These observations demonstrate that the loss of expression of LRP5 is not sufficient to prevent PA-mediated uptake of FP59 or LF. Similar tests were performed with MEFs from *Lrp6* ^+/+^ parental, *Lrp6* ^+/−^ heterozygous, and *Lrp6* ^−/−^ nullizygous mice. Each of these MEFs demonstrated equal sensitivity to treatment with combinations of PA plus FP59 (Figure 2A and 2B). Again, as an independent measure of PA-mediated entry, MEFs were treated with PA plus LF, and lysates were immunoblotted for N-terminal proteolysis of MEK1. *Lrp6* ^+/+^ parental, *Lrp6* ^+/−^ heterozygous, and *Lrp6* ^−/−^ nullizygous MEFs demonstrated equal MEK1 cleavage following treatment with PA plus LF (Figure 2C). These observations indicate that the loss of expression of *Lrp6* is not sufficient to prevent PA-mediated uptake of FP59 or LF. Collectively, these results demonstrate that neither *Lrp5* nor *Lrp6* is essential for PA-mediated uptake of FP59 or LF.

###  Neither *Lrp5* nor *Lrp6* Functions in a Receptor-Specific Fashion

The results discussed above indicate that PA can mediate entry of toxin into cells in the absence of either Lrp5 or Lrp6 and are at odds with a recent study in which an essential role for LRP6 in internalization and lethality of AnTx was reported [22]. In that study, the authors showed that *LRP6*-specific small interfering RNA (siRNA) and polyclonal antibodies raised against peptides corresponding to the extracellular domain of LRP6 could protect M2182 human prostate carcinoma cells and RAW264.7 murine macrophages from the lethal effects of FP59 or anthrax LeTx. One possible explanation for this discrepancy is that the different cell types used in these studies may differentially express ANTXR1 or ANTXR2 and that LRP6 acts in a receptor-specific fashion.

**Figure 3:**
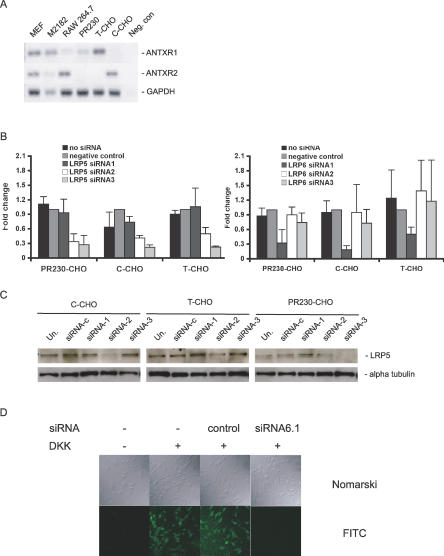
ANTXR Expression and siRNA Knockdown of LRP5 and LRP6 Reverse-transcription PCR was used to assess ANTXR expression in (A) M2182 prostate carcinoma, RAW264.7 macrophage, MEFs, and ANTXR-deficient PR230-CHO cells that were engineered to stably express either ANTXR1 (T-CHO) or ANTXR2 (C-CHO). PCR amplification of glyceraldehyde-3-phosphate dehydrogenase (GAPDH) is included as an mRNA control. (B) The efficacy with which LRP5- and LRP6-specific siRNA knocked down target gene expression was measured by real-time PCR. Results are presented as an average of three independent samples, each of which was run in duplicate. The error bars indicate the standard deviation about the mean. (C) The effect of Lrp5-specific siRNA and control siRNA (siRNAc) upon protein levels was assessed by immunoblotting with an antibody specific to Lrp5. Levels of α-tubulin are shown as a control for protein levels. Un., untreated. (D) The effect of Lrp6-specific siRNA and control siRNA upon Lrp6 protein levels was assessed by DKK-1 binding assays. Nomarski and fluorescence (FITC) images of cells are shown.

We used reverse-transcription PCR (RT-PCR) to compare the expresssion of *ANTXR* 1 and 2 in both the MEFs that we used, M2182 cells, and RAW264.7 cells. Interestingly, whereas MEFs uniformly expressed both *ANTXR*1 and *ANTXR*2, M2182 expressed *ANTXR*1 and undetectable levels of *ANTXR*2, and RAW264.7 cells expressed *ANTXR*2 and undetectable levels of *ANTXR*1 (Figure 3A). Similar results have been previously obtained for *ANTXR* expression in RAW264.7 cells [44]. This indicates that the cell lines used in the previous study and the MEF cells used in this study do indeed differentially express *ANTXR*.

**Figure 4:**
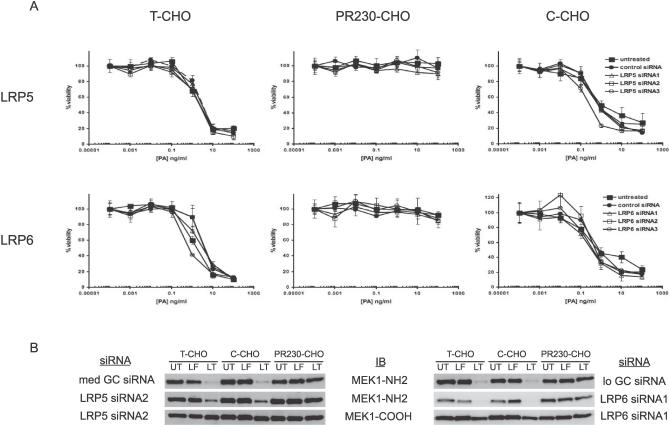
The Effect of LRP5 or LRP6 Knockdown upon PA-Dependent Uptake of FP59 and Anthrax LF (A) The effect of Lrp5 or Lrp6 knockdown on cell sensitivity to PA plus FP59 (1 ng/ml) was assessed in ANTXR-deficient PR230-CHO cells that were engineered to stably express either ANTXR1 (T-CHO) or ANTXR2 (C-CHO) using toxicity assays as described in Materials and Methods. Cell viability (oordinate) at the end of 24 h of incubation is plotted versus the concentration of PA (abcissa). Representative data from one of three experiments is presented. Error bars indicate standard deviation between quadruplicate samples, which were run pairwise on two separate plates. (B) As an independent indicator of PA-mediated entry into the siRNA-treated CHO cells, cleavage of MEK1 following treatment with medium alone (UT), LF alone, or lethal toxin (LT) was assessed by immunoblotting (IB) with antibodies that are specific for the NH_2_-terminus of MEK1. Only representative data for negative control siRNA (med GC and lo GC siRNA), Lrp5 siRNA2, and Lrp6 siRNA1 are shown, though identical results were obtained for Lrp5 siRNA 1 and 3 as well as Lrp6 siRNA 2 and 3. Immunoblots with an antibody against the carboxy-terminus of MEK1 (MEK1-COOH) are shown as a control for loading and protein degradation.

Based on these results, we speculated that PA-mediated internalization via LRP6 may work preferentially through either ANTXR1 or ANTXR2. Accordingly, we predicted that loss of *Lrp5* or *Lrp6* expression would disrupt PA-mediated uptake via one ANTXR but not the other. To test this hypothesis, we assayed the effects of siRNA inhibition of *Lrp5* and *Lrp6* expression upon PA-dependent uptake of FP59 in *ANTXR*-deficient PR230–Chinese hamster ovary (CHO) cells that were engineered to stably express either human *ANTXR*1 (T-CHO) or human *ANTXR*2 (C-CHO). Using RT-PCR, we confirmed previous observations [45,46] that these cell lines express neither *ANTXR*1 and *ANTXR*2, *ANTXR*1, nor *ANTXR*2, respectively (Figure 3A). By immunoblotting for the NH_2_-terminus of MEK1, we also confirmed that T-CHO and C-CHO, but not PR230-CHO, were capable of internalizing LF in a PA-dependent fashion (unpublished data; Figure 4B). To knock down *Lrp* expression in these cells, we tested three siRNA for *Lrp5* and three siRNA for *Lrp6*. Although these siRNAs were designed to inhibit mouse mRNA, we expect that hamster sequences will be highly homologous, if not identical; the hamster mRNA sequence obtained for regions of Lrp5 and Lrp6 that we used to design our PCR primers were 91%–95% identical to those published for mice (unpublished data). Using real-time PCR, we established that *Lrp5*-siRNA2 and *Lrp5*-siRNA3 were most effective in reducing *Lrp5* expression, and *Lrp6*-siRNA1 was most effective for reducing *Lrp6* expression (Figure 3B). Notably, the *Lrp6*-siRNA1 caused a similar level of mRNA inhibition as the siRNA used by Wei et al. [22]. Immunoblots with antibodies specific for Lrp5 showed that its levels were reduced in response to either siRNA-2 or siRNA-3, though the levels of protein did not strictly correlate with the level of mRNA expression (Figure 3C). Antibodies against Lrp6 did not work well in immunoblots (unpublished data), so we indirectly assayed Lrp6 protein expression by assaying the ability of siRNA-treated cells to bind the Lrp6 ligand DKK-1. Whereas treatment of PR230-CHO cells with Lrp6 siRNA-1 caused a clear reduction in DKK-1 binding, treatment with control siRNA did not cause any discernable loss of DKK-1 binding (Figure 3D). These results indicate that siRNA directed against either Lrp5 or Lrp6 can selectively reduce targeted mRNA and protein expression levels.

Regardless of the level of knockdown achieved, none of the siRNA had a demonstrable effect on PA-mediated uptake of FP59 (Figure 4A). In addition, none of the siRNA had any noticeable effect upon PA-mediated uptake of LF, as judged by immunoblotting for NH_2_-terminal epitopes of MEK1 (Figure 4B). These data indicate that PA-mediated internalization via either LRP5 or LRP6 does not work preferentially through either ANTXR1 or ANTXR2.

**Figure 5:**
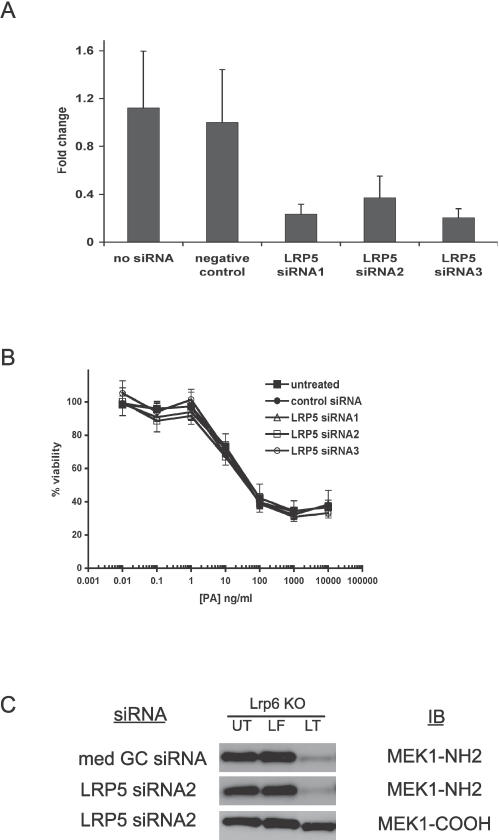
The Effect of Lrp5 Knockdown upon PA-Dependent Uptake of FP59 and Anthrax LF in Lrp6^−/−^ MEFs (A) The efficacy with which LRP5-specific siRNA knocked down target gene expression was measured by qPCR. Results are presented as an average of two independent samples, each of which was run in duplicate. The error bars indicate the standard deviation. (B) The effect of Lrp5 knockdown on cell sensitivity to PA plus FP59 (1 ng/ml) was assessed in Lrp6^−/−^ MEFs using toxicity assays as described in Materials and Methods. Cell viability (oordinate) at the end of 24 h of incubation is plotted versus the concentration of PA (abcissa). Error bars indicate standard deviation between two independent experiments, each of which was run in quadruplicate. (C) As an independent indicator of PA-mediated entry into the siRNA-treated CHO cells, cleavage of MEK1 was assessed by immunoblotting with antibodies that are specific for the NH_2_-terminus of MEK1. Only representative data for negative control siRNA (med GC) and LRP5 siRNA2 are shown, though identical results were obtained for LRP5 siRNA 1 and 3. An immunoblot with an antibody against the carboxy-terminus of MEK1 (MEK1-COOH) is shown as a control for loading and protein degradation.

Finally, we examined the possibility that Lrp5 or Lrp6 are functionally redundant and that PA-mediated internalization requires expression of either Lrp5 or Lrp6. MEFs isolated from *Lrp6* knockout mice were treated with *Lrp5*-specific siRNA and assayed for sensitivity to PA plus FP59 or LF. Using real–time PCR, we established that *Lrp5*-siRNA1, *Lrp5*-siRNA2, and *Lrp5*-siRNA3 were similarly effective in reducing *Lrp5* expression by approximately 66%–80% (Figure 5A). Despite this, reduced expression of Lrp5 in this Lrp6 null background had no effect upon sensitivity of MEFs to PA plus FP59 (Figure 5B) or upon LF cleavage of MEK1 (Figure 5C). Similar results were obtained when we treated *Lrp5* null MEFs with siRNA specific for *Lrp6,* or when we treated T-CHO cells with a combination of *Lrp5*-siRNA2 and *Lrp6*-siRNA1 (unpublished data). These results indicate that PA-mediated internalization by MEFs or CHO cells proceeds independently of the expression of Lrp5 and Lrp6.

## Discussion

Of the three AnTx proteins, PA is the central receptor-binding component, or B moiety, that delivers the catalytic effector molecules, LF or EF, to the cytosol [21]. Before it can do this, PA must bind a cell surface receptor. Cell surface receptors for PA (ANTXRs) have been identified [15,16]. The first receptor identified (ANTXR1) was a splice variant (sv2) of TEM8, a protein of unknown function that is up-regulated in colorectal cancer endothelium [47]. Young and colleagues now report that of three TEM8 splice variants tested, two (sv1 and sv2) function as an ANTXR, while the third (sv3) does not [16]. sv1 and sv2 differ only in the length of their cytoplasmic tails, but analysis of ANTXR1 deletion mutants indicates that this tail is dispensable for receptor function [45]. More recently, Scobie et al. [16] identified a second receptor (ANTXR2) encoded by capillary morphogenesis gene 2 (CMG2). As this name suggests, ANTXR2 is a protein which is up-regulated during endothelial cell morphogenesis [48]. Both ANTXR1 and ANTXR2 are ubiquitously expressed, making it likely that the participation of each is relevant to the pathology of anthrax.

Wei et al. [22] made a well-reasoned argument that LRP6 is essential for PA-dependent internalization of AnTx. Based on their initial findings, we further tested the requirement for not only Lrp6, but also Lrp5, in PA-dependent uptake of FP59 or LF. However, our results have directed us to the opinion that neither Lrp5 nor Lrp6 is essential for PA-dependent uptake into cells. This position is supported by three independent and compelling lines of evidence. First, mice with targeted deletions of *Lrp5* or *Lrp6* are as sensitive as wild-type mice to intravenous injections of anthrax LeTx. Second, wild-type MEFs and MEFs lacking expression of *Lrp5* or *Lrp6* are equally capable of internalizing either FP59 or LF in a PA-dependent fashion. Finally, knockdown of *Lrp5* or *Lrp6* with siRNA neither alters the sensitivity of CHO cells expressing ANTXR1 or ANTXR2 to FP59 nor prevents NH_2_-terminal proteolysis of MEK1 by LF. Thus, the available evidence does not support the hypothesis that either LRP5 or LRP6 plays an essential role in PA-mediated uptake.

We propose several alternative explanations for the discrepancy between our results and those of Wei et al. [22].

First, LRP6 may function in a species-specific fashion. Though Wei et al. [22] presented data from both human M2182 prostate carcinoma cells and mouse RAW264.7 macrophages, the protection conferred by siRNA in the latter was only modest (an approximately 2- to 3-fold increase in the IC_50_) when compared to that in the former (>100-fold increase in the IC_50_). Perhaps RAW264.7 cells, as well as MEF and CHO cells, express a splice variant of *Lrp6* that is resistant to siRNA treatment? This seems unlikely given the highly conserved nature of *LRP6;* LRP6 and Lrp6 share 97% protein sequence identity*.* Moreover, though the Ensembl browser (version 41, October 2006; http://www.ensembl.org) does predict a novel splice variant of human, but not mouse *Lrp5* containing an additional exon (2 base pair [bp]) between exons 13 and 14, splice variants of *Lrp6* have been not been characterized or predicted based on human or mouse genomic sequence. Alternatively, perhaps human ANTXRs are uniquely dependent upon LRP6 function. However, the CHO cells we used in this study expressed only human ANTXR1 or ANTXR2. Since these cells were equally sensitive to AnTx in the presence or absence of siRNA specific for Lrp5 or Lrp6, this indicates that 1) human ANTXR can bind and internalize AnTx in the absence of (human) LRP5 or LRP6, and 2) human ANTXRs do not have a general requirement for (murine) Lrp5 or Lrp6 in their function.

Second, LRP6 may function in a cell-specific manner. We cannot exclude this possibility. However, Lrp6 function is compromised even in heterozygous null mice since heterozygous expression genetically enhances a Wnt mutant phenotype [28]. Therefore, we argue that if LRP6 does function in a cell-specific manner, it is unlikely that sensitive cell types will play a significant role in the pathology of anthrax LeTx since heterozygous knockout mice are as sensitive to LeTx as their wild-type counterparts are. Thus, targeting of LRP6 will not likely prove useful in protecting against the effects of accumulated toxin during the late stages of anthrax disease when antibacterial methods normally are no longer of therapeutic value.

Third, LRP6 may function in a receptor-specific fashion. In support of this, our PCR analysis of the MEFs used in our study and the M2182 and RAW264.7 cells used by Wei et al. [22] indicate these cell types differentially express *ANTXR*1 and *ANTXR*2. Despite this, knockdown of either *Lrp5* or *Lrp6* by siRNA failed to protect CHO cells expressing one or the other receptor from PA-mediated entry of FP59 or LF. These results are inconsistent with the hypothesis that LRP5 or LRP6 functions in a receptor-specific fashion.

Fourth, it is possible that LRP5 and LRP6 are functionally redundant with regard to PA-mediated uptake. However, we observed that mouse and hamster cells deficient for both *Lrp5* and *Lrp6* expression are as sensitive to PA-mediated uptake as are control cells expressing both *Lrp5* and *Lrp6*. Further, since real-time PCR analysis of CHO cells (Figure 3) and M2182 cells (unpublished data) indicates that both cell types express *Lrp5* and *Lrp6,* the insensitivity of M2182 cells to PA-mediated toxicity following *Lrp6* knockdown is not likely explained by a deficiency in *Lrp5* expression. Finally, though the organization of the extracellular domains of LRP5 and LRP6 are similar to each other, they are markedly different from that of other LDL receptors [49]. So while we cannot exclude the possibility that LRP6 is functionally redundant with another LDL receptor, this possibility is remote.

Finally, the antisense screen used by Wei et al. [22] identified a human EST (image clone 285207) corresponding to an intronic region between exons 21 and 22 of *Lrp6*. Although the clone harboring this EST showed decreased levels of Lrp6 protein, the lack of a direct relationship between the EST and its apparent target *Lrp6* mRNA raises concerns regarding its specificity. Indeed, Wei and colleagues also reported that the same EST matches a sequence of the non-coding strand of an intron of a *Bcl-2*-like gene *(Bcl2L14)*. In contrast, for the *Lrp5* and *Lrp6* knockout mice used in this study, we can be reasonably assured that only *Lrp5* or *Lrp6* are targeted for inactivation, based on the original Southern blotting of mouse embryonic stem cell (ES) clones. Moreover, we may be reasonably assured that functional Lrp5 or Lrp6 is not expressed in these mice or cells derived from these mice. To generate *Lrp5* null mice, part of exon 1, including the signal peptide ATG, was replaced with an IRES-β-galactosidase reporter and MC1-neomycin phosphotransferase selection cassette. Thus, despite the fact that *Lrp5* ^−/−^ mice are viable, functional LRP5 cannot be expressed. To generate *Lrp6* null mice, a large insertion containing the splice acceptor of the mouse engrailed 2 gene, the transmembrane domain of rat CD4, and the β-galactosidase-neo reporter was inserted between exons 5 and 6. This insertion is therefore expected to generate a fusion construct encoding the first 457 of 1,370 extracellular domain amino acids of Lrp6, corresponding to the first YWTD β-propeller domain and an epidermal growth factor–like repeat of the extracellular domain. Significantly, this protein lacks the intracellular domain that Wei et al. [22] determined was essential for Lrp6 function in AnTx uptake.

In conclusion, using three independent approaches, we have failed to find evidence to support the hypothesis that either LRP5 or LRP6 plays an essential role in PA-mediated uptake. The more likely explanations for the discrepancies between these reports is that either the EST identified by Wei et al. [22] is not specific for *LRP6,* or the role of LRP6 in PA-mediated uptake is cell-type specific. However, our results should not be interpreted as an indication that an association of ANTXR with PA is sufficient for cellular internalization. Indirect evidence suggests that other membrane-associated proteins may also play a role in AnTx uptake. By chemically cross-linking associated surface proteins, Escuyer and Collier [50] estimated the molecular weight of PA complexed with its receptor at approximately 170 kDa. Since the molecular weights of activated PA, ANTXR1 sv1, ANTXR1 sv2, and ANTXR2 are 63 kDa, 63 kDa, 41 kDa, and 43 kDa, respectively, this indicates that either PA does not bind ANTXR in a 1:1 ratio, or other as yet unidentified proteins are present in this complex.

## Materials and Methods

### *Lrp5*- and *Lrp6*-deficient mice.

*Lrp5*-deficient mice were generated as described in [42]. *Lrp6*-deficient mice (a gift of W. Skarnes) have been described previously [28]. The knockout mice in this report are maintained on a C57BL background. The genotypes of all mice used in this study were confirmed by PCR analysis of genomic DNA. All experiments were performed in compliance with the guiding principles of the *Guide for the Care and Use of Animals* by the National Academy of Sciences Institute of Laboratory Animal Resources Commission on Life Sciences. In addition, all procedures were approved before use by the Institutional Animal Care and Use Committee of the Van Andel Research Institute.

### Protein expression and purification.

PA and LF were expressed in Bacillus anthracis (BH445) and purified essentially as described by Park and Leppla [51]. The concentration of each protein was estimated using the bicinchoninic acid method [52] and by densitometric analyses of Coomassie Blue-stained polyacrylamide gels.

### In vivo toxicity assays.

To assess the requirement for Lrp5 and Lrp6 in response to LeTx in vivo, 6- to 20-wk-old (male) wild-type, heterozygous, and knockout *Lrp5* and *Lrp6* mice were injected daily via the tail vein with 50 μl of Hank's buffered salt solution containing PA (50 ug) and LF (10 ug). The animals were weighed daily and monitored for signs of stress or discomfort.

### Cell culture and reagents.

J774A.1 cells (obtained from the American Type Culture Collection, http://www.atcc.org) as well as MEFs obtained from wild-type, heterozygous, and knockout Lrp5 and Lrp6 mice were cultured in Dulbecco's Modified Eagle Medium supplemented with 10% fetal bovine serum and 1% penicillin/streptomycin. M2182 cells were cultured in RPMI1640 supplemented as described previously [53]. All cell lines were maintained at 37 °C in a humidified 5% CO_2_ incubator. A spontaneous ANTXR-deficient CHO cell mutant (PR230-CHO) as well as PR230-CHO stably transfected with human ANTXR1 (T-CHO) or human ANTXR2 (C-CHO) expression vectors [45] were cultured in Ham's F-12 medium supplemented with 10% fetal bovine serum and 1% penicillin/streptomycin.

### Cytotoxicity assays.

Cells were grown in 96-well plates to 70% confluence. Cells were treated with culture medium containing PA plus FP59 at the concentrations indicated and incubated 20 h at 37 °C. At the end of the experiment, cell viability was determined using the CellTiter 96 Aqueous Non-Radioactive Cell Proliferation Assay (Promega, http://www.promega.com) according to the manufacturer's instructions.

### MEK cleavage assays.

To assay MEK1 cleavage, we made lysates of murine cells which had been incubated for 4 h with 0.1 μg/ml PA and 0.01 μg/ml LF. Lysates were separated by denaturing SDS-PAGE and immunoblotted with antibodies raised against the NH_2_-terminus (07–641, 1:1000; Upstate Biotechnology, http://www.upstate.com) or the COOH-terminus (sc-219, 1:1000; Santa Cruz Biotechnology, http://www.scbt.com) of MEK1. Immunoblots were stripped and re-probed for α-tubulin (T9026, 1:1000; Sigma, http://www.sigmaaldrich.com) for a loading control.

### Genotypic analysis of embryonic fibroblasts.

DNA was prepared from embryonic fibroblasts using an AutoGenprep 960 automated DNA isolation system (AutoGen, http://www.autogen.com). The sequences of the PCR primers used in this study are listed in Table S1. PCR of the *Lrp5* alleles was carried out in 25 μl of total volume containing 2.5 μl of 10× PCR buffer (Invitrogen, http://www.invitrogen.com), 2.0 μl of DMSO, 1.0 μl of 50 mM MgCl_2_, 0.625 mM of each nucleotide, 1 U of Taq polymerase (Invitrogen), a 6.25-μg/ml concentration of primers *LRP5* F or Neo F1, and a 12.5-μg/ml concentration of primer LRP5 3′-targeted (common primer) to detect a 430-bp fragment of the wild-type allele and/or a 1,000-bp fragment of the mutant allele. Samples were amplified for 34 cycles (94 °C for 1 min, 57.8 °C for 1 min, and 72 °C for 1 min). PCR of the wild-type *Lrp6* allele was carried out as above except with no DMSO and using the LRP6 7757-S primer and the LRP6 8085-AS primer (to detect a 325-bp fragment). Samples were amplified for 30 cycles (94 °C for 45 s, 56.5 °C for 45 s, and 72 °C for 1 min). PCR of the mutant LRP6 allele was carried out as above in 20 μl total volume and no DMSO using the LRP6 7757-S primer and the pGT1.8TM-1388 AS primer (to detect a 586-bp fragment). Samples were amplified for 30 cycles (94 °C for 45 s, 56 °C for 45 s, and 72 °C for 1 min). PCR products were visualized by ethidium bromide staining in 1.0% agarose gels.

### PCR analysis of *ANTXR* and *Lrp* 5/6 expression.

RNA was isolated using the TRIzol (Invitrogen) method. Briefly, cells were lysed in 10 ml TRIzol and RNA was extracted with chloroform using a 15-ml phase-lock tube (Eppendorf, http://www.eppendorf.com). RNA was precipitated with isopropyl alcohol, washed in 75% ethanol, and pellets were air dried. Pellets were re-suspended in nuclease-free water. Reverse-transcription PCR of *ANTXR*1 was carried out in 50 μl total volume containing 5 μl of 10× PCR buffer (Invitrogen), 1.5 μl of 50 mM MgCl_2_, 0.2 mM of each nucleotide, 0.4 μl of Taq Polymerase, and 0.20-μM concentration of primers. To detect murine *ANTXR1,* we used the primers mANTXR1 F and mANTXR1 R (to detect a 258-bp fragment). To detect human *ANTXR1,* we used the primers hANTXR1 F and hANTXR1 R to detect a 256-bp fragment. Samples were amplified for 36 cycles (94 °C for 45 s, 55 °C for 30 s, and 72 °C for 1.5 min). RT-PCR of *ANTXR2* was carried out as described above except that the following primers were used: mouse mANTXR2 F and mANTXR2 R to detect a 364-bp fragment, and human hANTXR2 F and hANTXR2 R to detect a 344-bp fragment. PCR products were visualized by SYBR Safe DNA gel stain (Invitrogen) in 1.0% agarose gels. The identity of the PCR products was confirmed by DNA sequencing.

RT-PCR of *Lrp5* and *Lrp6* from CHO cells was carried out essentially as described. The following primers were used: Lrp5 bp336 F and Lrp5 bp895 R (to detect a 559-bp fragment), Lrp5 bp2230 F and Lrp5 bp2699 R (to detect a 469-bp fragment), and ham Lrp6 F and ham Lrp6 R. Samples were amplified for 36 cycles (94 °C for 45 s, 60 °C (or 55 °C for Lrp6) for 30 s, and 68 °C for 3 min). PCR products were visualized by SYBR Safe DNA gel stain (Invitrogen) in 1.0% agarose gels. The identity of the PCR products was confirmed by DNA sequencing. These partial sequences were used later to generate primers for real-time PCR.

### Inhibition of Lrp5 and Lrp6 expression.

To inhibit *Lrp5* and *Lrp6* gene expression in CHO cells and MEFs, we utilized Stealth RNAi to knock down mouse *Lrp5* and *Lrp6* (Invitrogen). Stealth RNAi low GC and medium GC duplexes were used as negative controls for *Lrp6* and *Lrp5,* respectively. Cells were transfected at 30% confluence using Lipofectamine 2000 according to the manufacturer's protocol (Invitrogen) with a final concentration of 40 nM siRNA. After 48 h incubation, cells were treated with culture medium containing PA plus FP59 at the concentrations indicated for 3 h, after which the toxin-containing medium was removed, fresh medium added, and cells were incubated for an additional 16 h at 37 °C. At the end of the experiment, cell viability was determined using the CellTiter 96 Aqueous Non-Radioactive Cell Proliferation Assay as described above. Additionally, to analyze the effect of siRNA treatment on the function of LeTx, treated cells were subjected to LeTx (0.1 μg/ml PA and 0.01 μg/ml LF) or LF alone (0.01 μg/ml LF) for 4 h, after which cells were either collected for analysis of MEK cleavage or for verification of mRNA knockdown.

### Real-time PCR of Lrp5 and Lrp6.

To analyze Lrp5 or Lrp6 mRNA levels after siRNA treatment, quantitative real-time PCR was performed using TaqMan One-Step RT-PCR Master Mix (Applied Biosystems, http://www.appliedbiosystems.com) and detected using the Applied Biosystems 7300 Real-Time PCR System. Analysis of the level of 18S ribosomal RNA (18S rRNA) in each sample was performed as an internal loading control. Primers and probes were designed based on partial mRNA sequence for hamster Lrp5 and Lrp6 using Primer Express software (Applied Biosystems). Real-time RT-PCR was carried out under the following conditions: 2× Master Mix without UNG (1× final concentration), 40× MultiScribe and RNase Inhibitor Mix (0.25 U/uL and 0.4 U/uL, respectively, final concentration), 200 nM (Lrp5/6) or 50 nM (18S rRNA) forward (ham F) and reverse (ham R) primers, 100 nM (Lrp5/6) or 50 nM (18S rRNA) TAMRA probe, and 100 ng (Lrp5/6) or 10 ng (18S rRNA) of total RNA. The reverse-transcription step was performed at 48 °C for 30 min, after which PCR was performed at 95 °C for 10 min, followed by 40 cycles of 95 °C for 15 s and 60 °C for 1 min. Standard curves were performed for each gene to be analyzed, and used to determine the relative value of mRNA per gene. The value of Lrp5 or Lrp6 mRNA was normalized to endogenous 18S rRNA and expressed in terms of a fold-change over the negative control. All samples were run in triplicate for each reaction, and all experiments were performed and analyzed a total of three times.

### DKK-1 binding assays.

Cells were seeded on chambered glass slides (Lab Tek II; Nalge Nunc International, http://www.nalgenunc.com) and siRNA transfections were performed on PR230-CHO cells as described above. After 48 h siRNA treatment, the slides were placed on ice and the chambers washed with ice-cold culture medium. Following this, cells were incubated with ice-cold culture medium supplemented with 100 pM His-tagged DKK-1 (R&D Systems, http://www.rndsystems.com) or culture medium alone for 2 h. Cells were then washed three times with ice-cold PBS and fixed with 4% formalin for 15 min. Cells were rinsed with PBS and blocked for 40 min with 10% FBS in PBS. A FITC-conjugated anti-His antibody (Invitrogen, 1:1000) was used to detect DKK-1. Cells were then washed twice with PBS + 0.1% Triton-X100, mounted, and imaged using a Zeiss 510 confocal microscope and Zeiss image acquisition software (http://www.zeiss.com). All images were acquired using identical parameters.

## Supporting Information

Table S1 List and Sequences of Primers Used in This Study (61 KB DOC)

Video S1 J774A.1 Macrophages Treated with PA Plus LF J774A.1 murine macrophages treated with PA plus LF undergo abrupt lysis and cell death within 4 h of treatment. Images acquired with the assistance of Art Alberts, Van Andel Research Institute.
(1.1 MB MOV)

### Accession Number

The GenBank (http://www.ncbi.nlm.nih.gov/Genbank/index.html) accession number for image clone 285207 is N66273.

## Abbreviations

AnTx: anthrax toxin
ANTXR: anthrax toxin receptor
bp: base pair
CHO: Chinese hamster ovary
EF: edema factor
EST: expressed sequence tag
LDL: low-density lipoprotein
LeTx: lethal toxin
LF: lethal factor
MEF: murine embryonic fibroblast
MEK: mitogen activated protein kinase/extracellular signal-regulated kinase kinase
OPPG: osteoporosis pseudoglioma syndrome
PA: protective antigen
RT-PCR: reverse-transcription PCR
siRNA: small interfering RNA

## References

[ppat-0030027-b001] Bloom WL, McGhee WJ, Cromartie WJ, Watson DW (1947). Studies on infection with Bacillus anthracis. VI. Physiological changes in experimental animals during the course of infection with B. anthracis. J Infect Dis.

[ppat-0030027-b002] Smith H, Keppie J (1954). Observations on experimental anthrax: Demonstration of a specific lethal factor produced in vivo by Bacillus anthracis. Nature.

[ppat-0030027-b003] Klimpel KR, Molloy SS, Thomas G, Leppla SH (1992). Anthrax toxin protective antigen is activated by a cell surface protease with the sequence specificity and catalytic properties of furin. Proc Natl Acad Sci U S A.

[ppat-0030027-b004] Molloy SS, Bresnahan PA, Leppla SH, Klimpel KR, Thomas G (1992). Human furin is a calcium-dependent serine endoprotease that recognizes the sequence Arg-X-X-Arg and efficiently cleaves anthrax toxin protective antigen. J Biol Chem.

[ppat-0030027-b005] Singh Y, Leppla SH, Bhatnagar R, Friedlander AM (1989). Internalization and processing of Bacillus anthracis lethal toxin by toxin-sensitive and -resistant cells. J Biol Chem.

[ppat-0030027-b006] Petosa C, Collier RJ, Klimpel KR, Leppla SH, Liddington RC (1997). Crystal structure of the anthrax toxin protective antigen. Nature.

[ppat-0030027-b007] Duesbery NS, Webb CP, Leppla SH, Gordon VM, Klimpel KR (1998). Proteolytic inactivation of MAP-kinase-kinase by anthrax lethal factor. Science.

[ppat-0030027-b008] Vitale G, Pellizzari R, Recchi C, Napolitani G, Mock M (1998). Anthrax lethal factor cleaves the N-terminus of MAPKKs and induces tyrosine/threonine phosphorylation of MAPKs in cultured macrophages. Biochem Biophys Res Commun.

[ppat-0030027-b009] Pellizzari R, Guidi-Rontani C, Vitale G, Mock M, Montecucco C (1999). Anthrax lethal factor cleaves MKK3 in macrophages and inhibits the LPS/IFNgamma-induced release of NO and TNFalpha. FEBS Lett.

[ppat-0030027-b010] Vitale G, Bernardi L, Napolitani G, Mock M, Montecucco C (2000). Susceptibility of mitogen-activated protein kinase kinase family members to proteolysis by anthrax lethal factor. Biochem J.

[ppat-0030027-b011] Chopra AP, Boone SA, Liang X, Duesbery NS (2003). Anthrax lethal factor proteolysis and inactivation of MAPK kinase. J Biol Chem.

[ppat-0030027-b012] Bardwell AJ, Abdollahi M, Bardwell L (2004). Anthrax lethal factor-cleavage products of MAPK (mitogen-activated protein kinase) kinases exhibit reduced binding to their cognate MAPKs. Biochem J.

[ppat-0030027-b013] Singh Y, Liang X, Duesbery NS, Proft T (2005). Pathogenesis of *Bacillus anthracis:* The role of anthrax toxins. Microbial toxins: Molecular and cellular biology.

[ppat-0030027-b014] Friedlander AM (1986). Macrophages are sensitive to anthrax lethal toxin through an acid-dependent process. J Biol Chem.

[ppat-0030027-b015] Bradley KA, Mogridge J, Mourez M, Collier RJ, Young JA (2001). Identification of the cellular receptor for anthrax toxin. Nature.

[ppat-0030027-b016] Scobie HM, Rainey GJ, Bradley KA, Young JA (2003). Human capillary morphogenesis protein 2 functions as an anthrax toxin receptor. Proc Natl Acad Sci U S A.

[ppat-0030027-b017] Mogridge J, Cunningham K, Collier RJ (2002). Stoichiometry of anthrax toxin complexes. Biochemistry.

[ppat-0030027-b018] Leppla SH, Freer AA (1991). The anthrax toxin complex. Sourcebook of bacterial protein toxins.

[ppat-0030027-b019] Milne JC, Furlong D, Hanna PC, Wall JS, Collier RJ (1994). Anthrax protective antigen forms oligomers during intoxication of mammalian cells. J Biol Chem.

[ppat-0030027-b020] Gordon VM, Leppla SH, Hewlett EL (1988). Inhibitors of receptor-mediated endocytosis block the entry of Bacillus anthracis adenylate cyclase toxin but not that of Bordetella pertussis adenylate cyclase toxin. Infect Immun.

[ppat-0030027-b021] Leppla SH (1982). Anthrax toxin edema factor: A bacterial adenylate cyclase that increases cyclic AMP concentrations of eukaryotic cells. Proc Natl Acad Sci U S A.

[ppat-0030027-b022] Wei W, Lu Q, Chaudry GJ, Leppla SH, Cohen SN (2006). The LDL receptor-related protein LRP6 mediates internalization and lethality of anthrax toxin. Cell.

[ppat-0030027-b023] Dong Y, Lathrop W, Weaver D, Qiu Q, Cini J (1998). Molecular cloning and characterization of LR3, a novel LDL receptor family protein with mitogenic activity. Biochem Biophys Res Commun.

[ppat-0030027-b024] Hey PJ, Twells RC, Phillips MS, Yusuke N, Brown SD (1998). Cloning of a novel member of the low-density lipoprotein receptor family. Gene.

[ppat-0030027-b025] Brown SD, Twells RC, Hey PJ, Cox RD, Levy ER (1998). Isolation and characterization of LRP6, a novel member of the low density lipoprotein receptor gene family. Biochem Biophys Res Commun.

[ppat-0030027-b026] Tamai K, Semenov M, Kato Y, Spokony R, Liu C (2000). LDL-receptor-related proteins in Wnt signal transduction. Nature.

[ppat-0030027-b027] van Amerongen R, Berns A (2006). Knockout mouse models to study Wnt signal transduction. Trends Genet.

[ppat-0030027-b028] Pinson KI, Brennan J, Monkley S, Avery BJ, Skarnes WC (2000). An LDL-receptor-related protein mediates Wnt signalling in mice. Nature.

[ppat-0030027-b029] Wehrli M, Dougan ST, Caldwell K, O'Keefe L, Schwartz S (2000). *arrow* encodes an LDL-receptor-related protein essential for Wingless signalling. Nature.

[ppat-0030027-b030] Kato M, Patel MS, Levasseur R, Lobov I, Chang BH (2002). Cbfa1-independent decrease in osteoblast proliferation, osteopenia, and persistent embryonic eye vascularization in mice deficient in Lrp5, a Wnt coreceptor. J Cell Biol.

[ppat-0030027-b031] Fujino T, Asaba H, Kang MJ, Ikeda Y, Sone H (2003). Low-density lipoprotein receptor-related protein 5 (LRP5) is essential for normal cholesterol metabolism and glucose-induced insulin secretion. Proc Natl Acad Sci U S A.

[ppat-0030027-b032] Steichen-Gersdorf E, Gassner I, Unsinn K, Sperl W (1997). Persistent hyperplastic primary vitreous in a family with osteoporosis-pseudoglioma syndrome. Clin Dysmorphol.

[ppat-0030027-b033] Gong Y, Vikkula M, Boon L, Liu J, Beighton P (1996). Osteoporosis-pseudoglioma syndrome, a disorder affecting skeletal strength and vision, is assigned to chromosome region 11q12–13. Am J Hum Genet.

[ppat-0030027-b034] De Paepe A, Leroy JG, Nuytinck L, Meire F, Capoen J (1993). Osteoporosis-pseudoglioma syndrome. Am J Med Genet.

[ppat-0030027-b035] Capoen J, De Paepe A, Lauwers H (1993). The osteoporosis pseudoglioma syndrome. J Belge Radiol.

[ppat-0030027-b036] Gong Y, Slee RB, Fukai N, Rawadi G, Roman-Roman S (2001). LDL receptor-related protein 5 (LRP5) affects bone accrual and eye development. Cell.

[ppat-0030027-b037] Van Wesenbeeck L, Cleiren E, Gram J, Beals RK, Benichou O (2003). Six novel missense mutations in the LDL receptor-related protein 5 (LRP5) gene in different conditions with an increased bone density. Am J Hum Genet.

[ppat-0030027-b038] Van Hul E, Gram J, Bollerslev J, Van Wesenbeeck L, Mathysen D (2002). Localization of the gene causing autosomal dominant osteopetrosis type I to chromosome 11q12–13. J Bone Miner Res.

[ppat-0030027-b039] Little RD, Carulli JP, Del Mastro RG, Dupuis J, Osborne M (2002). A mutation in the LDL receptor-related protein 5 gene results in the autosomal dominant high-bone-mass trait. Am J Hum Genet.

[ppat-0030027-b040] Boyden LM, Mao J, Belsky J, Mitzner L, Farhi A (2002). High bone density due to a mutation in LDL-receptor-related protein 5. N Engl J Med.

[ppat-0030027-b041] Babij P, Zhao W, Small C, Kharode Y, Yaworsky PJ (2003). High bone mass in mice expressing a mutant LRP5 gene. J Bone Miner Res.

[ppat-0030027-b042] Holmen SL, Giambernardi TA, Zylstra CR, Buckner-Berghuis BD, Resau JH (2004). Decreased BMD and limb deformities in mice carrying mutations in both Lrp5 and Lrp6. J Bone Miner Res.

[ppat-0030027-b043] Arora N, Klimpel KR, Singh Y, Leppla SH (1992). Fusions of anthrax toxin lethal factor to the ADP-ribosylation domain of *Pseudomonas* exotoxin A are potent cytotoxins which are translocated to the cytosol of mammalian cells. J Biol Chem.

[ppat-0030027-b044] Banks DJ, Barnajian M, Maldonado-Arocho FJ, Sanchez AM, Bradley KA (2005). Anthrax toxin receptor 2 mediates Bacillus anthracis killing of macrophages following spore challenge. Cell Microbiol.

[ppat-0030027-b045] Liu S, Leppla SH (2003). Cell surface tumor endothelium marker 8 cytoplasmic tail-independent anthrax toxin binding, proteolytic processing, oligomer formation, and internalization. J Biol Chem.

[ppat-0030027-b046] Abi-Habib RJ, Urieto JO, Liu S, Leppla SH, Duesbery NS (2005). BRAF status and mitogen-activated protein/extracellular signal-regulated kinase kinase 1/2 activity indicate sensitivity of melanoma cells to anthrax lethal toxin. Mol Cancer Ther.

[ppat-0030027-b047] St Croix B, Rago C, Velculescu V, Traverso G, Romans KE (2000). Genes expressed in human tumor endothelium. Science.

[ppat-0030027-b048] Bell SE, Mavila A, Salazar R, Bayless KJ, Kanagala S (2001). Differential gene expression during capillary morphogenesis in 3D collagen matrices: Regulated expression of genes involved in basement membrane matrix assembly, cell cycle progression, cellular differentiation and G-protein signaling. J Cell Sci.

[ppat-0030027-b049] He X, Semenov M, Tamai K, Zeng X (2004). LDL receptor-related proteins 5 and 6 in Wnt/beta-catenin signaling: Arrows point the way. Development.

[ppat-0030027-b050] Escuyer V, Collier RJ (1991). Anthrax protective antigen interacts with a specific receptor on the surface of CHO-K1 cells. Infect Immun.

[ppat-0030027-b051] Park S, Leppla SH (2000). Optimized production and purification of Bacillus anthracis lethal factor. Protein Expr Purif.

[ppat-0030027-b052] Smith PK, Krohn RI, Hermanson GT, Mallia AK, Gartner FH (1985). Measurement of protein using bicinchoninic acid. Anal Biochem.

[ppat-0030027-b053] Jackson-Cook C, Bae V, Edelman W, Brothman A, Ware J (1996). Cytogenetic characterization of the human prostate cancer cell line P69SV40T and its novel tumorigenic sublines M2182 and M15. Cancer Genet Cytogenet.

