# Supplementary material for: LRP5 and LRP6 Are Not Required for Protective Antigen–Mediated Internalization or Lethality of Anthrax Lethal Toxin
Source: PLoS Pathog. 2007 Mar 2;3(3):e27. doi: 10.1371/journal.ppat.0030027 (PMC1808072; doi:10.1371/journal.ppat.0030027)
Supplement: Supplementary file 1 [file ppat.0030027.st001.doc]

**Table S1**

1. List and sequences of primers used in this study.

| **primer name** | **primer sequence** |
| --- | --- |
| *LRP5* 3’-targeted | 5’-GCTTCCATTTTAGGCCTGG-3’ |
| *LRP5* F | 5’-CTTCATCCAGTCTTGGAGC-3 |
| Neo F1 | 5’-ACAACAGACAATCGGCTGC-3’ |
| *LRP6* 7757-S | 5’-CAGTTAAGTATTGTGAGAAGAGAGCA-3’ |
| *LRP6* 8085-AS | 5’-TCCAAACTACAAGCCCTGCACT-3’ |
| pGT1.8TM-1388 AS | 5’- CGTTTGCATTTGTTTTTCTCC-3’ |
| mANTXR1 F | 5’-GAACAGATCCGACAAGGCCTAGAAG-3’ |
| mANTXR1 R | 5’-GAACAGATCCGACAAGGCCTAGAAG-3’ |
| mANTXR2 F | 5’-CTGACAGAGAGATTTGTGAGC-3’ |
| mANTXR2 R | 5’-GCAATTCTTTCCAGCTGA-3’ |
| hANTXR1 F | 5’-GAACAAATCCGTCAAGGCCTAGAAG -3’ |
| hANTXR1 R | 5’-TCATTGAAATCTTTCACACCAAC-3’ |
| hANTXR2 F | 5’-CTTGCGGAGAGATTTGTGAGC-3’ |
| hANTXR2 R | 5’-GCAATTCTTTCAAGCTG-3’ |
| muGAPDH F | 5’-TGAAGGTCGGTGTGAACGGATTTGGC-3’ |
| muGAPDH R | 5’-TAGTGGGGTCTCGCTCCTGGAAGATG-3’ |
| LRP5 bp336 F | 5’-TGGACAGATGTGAGCGAGGAGG-3’ |
| LRP5 bp895 R | 5’-TTGTTGCAGGCGTGGATGGAGC-3’ |
| LRP5 bp2230 F | 5’-AGCACGTGATTGAGTTTGG-3’ |
| LRP5 bp2699 R | 5’-ATGCAGGTTCCAGTCAGT-3’ |
| ham LRP6 F | 5’-GGCCCCTTTGTTGCTTTAT-3’ |
| ham LRP6 R | 5’-ATTTCACGCAGACCCTCG-3’ |
| LRP6 ham TaqMan | 5’-6FAMTGACCCTGGATTATGAGGAACGGATAMRA-3’ |
| LRP6 ham R | 5’-TTTGCATCTGCCCAATAAAGC-3’ |
| LRP6 ham F | 5’-TGAAATTTACTGGCCAAATGGA-3’ |
| LRP5 ham TaqMan | 5’-6FAMAGGACCTGGACCAGCCAAGGGCTAMRA-3’ |
| LRP5 ham R | 5’-TGCAGGATCCAGGGCAAT-3’ |
| LRP5 ham F | 5’-TCCCGCAAGGTTCTCTTCTG-3’ |
